# Food tax policies in Pacific Island Countries and Territories: systematic policy review

**DOI:** 10.1017/S1368980023002914

**Published:** 2023-12-21

**Authors:** Emilee Walby, Amanda C Jones, Moira Smith, Elisiva Na’ati, Wendy Snowdon, Andrea M Teng

**Affiliations:** 1 University of Otago Wellington, PO Box 7343, Newtown, Wellington, 6242, New Zealand; 2 Health Promotion Agency, Wellington, New Zealand; 3 Pacific Community, Suva, Fiji; 4 Globe Centre for Preventive Health and Nutrition, Deakin University, Geelong, VIC, Australia

**Keywords:** Food tax, Excise, Import tariff, Pacific, Unhealthy food

## Abstract

**Objective:**

To systematically identify and review food taxation policy changes in Pacific Island Countries and Territories (PICTs).

**Design:**

Food taxation polices, regarding excise taxes and tariffs applied from 2000 to 2020 in twenty-two PICTs, and their key characteristics were reviewed. The search was conducted using databases, government legal repositories and broad-based search engines. Identified documents for screening included legislation, reports, academic literature, news articles and grey literature. Key informants were contacted from each PICT to retrieve further data and confirm results. Results were analysed by narrative synthesis.

**Setting:**

Noncommunicable diseases (NCD) are the leading cause of premature death in PICTs and in many jurisdictions globally. An NCD crisis has been declared in the Pacific, and food taxation policy has been recommended to address the dietary risk factors associated with. Progress is unclear.

**Results:**

Of the twenty-two PICTs included in the study, fourteen had food taxation policies and five introduced excise taxes. Processed foods, sugar and salt were the main target of excise taxes. A total of eighty-four food taxation policy changes were identified across all food groups. There was a total of 279 taxes identified by food group, of which 85 % were tariffs and 15 % were excise taxes. Individual tax rates varied substantially. The predominant tax design was ad valorem, and this was followed by volumetric.

**Conclusions:**

A quarter of PICTs have introduced food excise taxes from 2000 to 2020. Further excise taxes, specifically tiered or nutrient-specific designs, could be introduced and more systematically applied to a broader range of unhealthy foods.

Pacific Island Countries and Territories (PICTs) have some of the highest rates of noncommunicable diseases (NCDs) globally^(1)^. CVD, diabetes, cancer and chronic respiratory diseases are widely prevalent^(2,3)^, with up to 90 % of deaths in the region’s countries attributable to NCDs^(4)^. The NCD-related challenges facing the PICTs are similar among the twenty-two nations, and the growth of NCDs across the region has been influenced by rapid dietary changes. Many PICTs are dependent on food imports, making up around 40 % to 50 % of dietary intake in several PICTs and more than 80 % in Palau (in the 2000s)^(5)^, but these levels have likely increased. It has been recognised that dietary policies are crucial to addressing the NCD crisis in PICTs, and in the last few decades, these countries have produced a broad suite of plans and measures to address the NCD crisis in the region^(6,7)^.

The use of food taxation policy has been as shown to be an effective fiscal instrument when aiming to adjust people’s consumption preferences^(8–12)^. The types of taxation that are used in targeted food taxation mechanisms are typically import tariffs and excise taxes (Table 1).

**Table 1:** Tax types in this study

Tax type	Definition
Import tariff (customs duty)	Tariffs are applied to goods when they are imported into the country. The level of tax is applied according to specific tariff codes on individual food products and usually is calculated as a percentage of the good’s import value (ad valorem).
Excise tax (health levy)	Excise taxes are generally applied to both imported and domestically produced goods. Excise tends to be targeted at specific goods for health and other public good reasons. For example, alcohol and tobacco taxes are usually excise taxes. The level of tax is usually calculated based on the good’s volume or weight (volumetric) or nutrient content, e.g. sugar content (nutrient-specific). Some excise taxes only apply when a good crosses a given threshold, e.g. at a given sugar concentration (tiered).

Studies show that taxation policies on sugar-sweetened beverages have been widely implemented in PICTs^(13,14)^, but systematic identification of food taxation policies and their characteristics was not currently available^(6,15)^. An inventory of current taxation policies for each PICT and their changes over time can provide evidence of the food taxation policies that have been introduced and their key features. A baseline set of data can assist in identifying opportunities for policy updates to improve nutrition outcomes. Alongside this, PICTs’ use of food taxation policy provides evidence of the action taken by Low Middle-Income Countries (LMIC) in championing the WHO recommendations^(16)^ of implementing food taxation policies to address NCD. This foundational knowledge can support the use of taxation policy as a tool in health promotion to address the NCD crisis. In addition, it can add to existing knowledge about food taxes and tariffs in the context of Small Island Developing States.

The aim of this study was to systematically review food tax policies, that is any tariff or excise tax on food that was introduced or modified by a PICT from 2000 to 2020.

## Methods

### Study design and scope

A systematic search was used to generate an inventory of enacted taxation policies applied to foods in PICTs and to review their characteristics. The unit of analysis was each individual taxation change.

### Inclusion and exclusion criteria

**Fig. 1:**
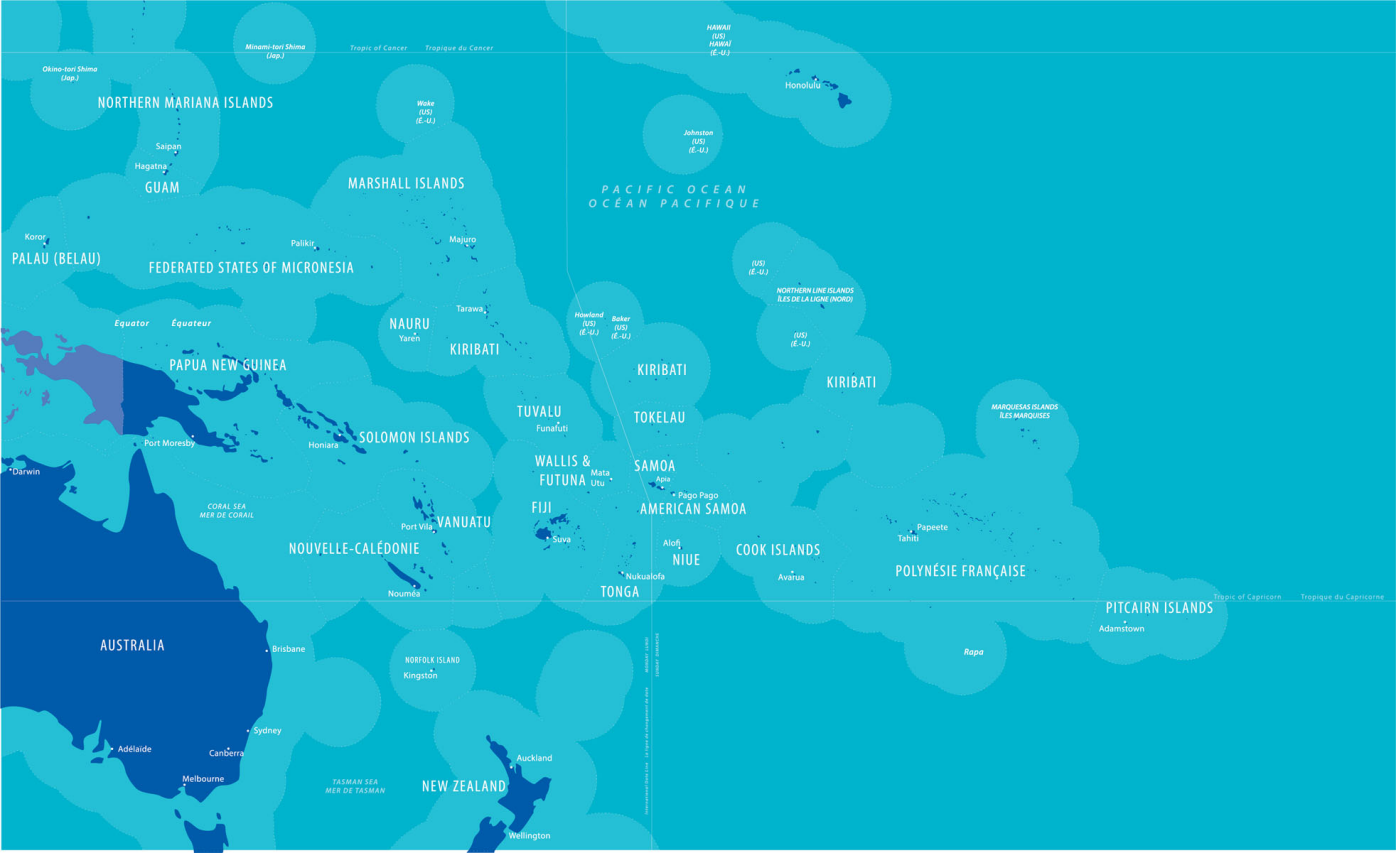
Pacific Island Countries and Territories. Pacific Community (SPC) Publications (http://www.paclii.org/maps/)

In scope were policies from twenty-two PICTs (Fig. 1)^(17)^; larger countries in the Pacific region were excluded (i.e. New Zealand and Australia) with the focus here on Small Island Developing States. Included taxation policies were excise taxes and tariffs on foods implemented from January 2000 to December 2020. Taxes were included if they were variably applied to groups of foods or specific food products. Taxes could be applied to locally produced foods, imported foods or both and could be based on a product’s value, volume or content (i.e. ad valorem, volumetric, tiered and nutrient-specific taxes, Table 1). This study focussed on taxes applied at a national level only. Taxes were excluded if they were applied at a flat rate across *all* foods. Taxation policies that applied tax exemptions to specific regions or industries were also excluded. Subsidies were not explicitly analysed.

English and French language taxation policies were both in-scope. Pacific languages were excluded as versions of these policies were also published in English.

### Search strategy and screening

Four search approaches were used to source taxation policies: (1) the Pacific Islands Legal Information Institute (PacLII) database, (2) large search engines (first fifty results of each search were examined) namely Google, Google Scholar, Factiva and Scopus, (3) available PICTs Government legislation repositories and (4) contact with key informants to provide any further data or advice. Searches involved identifying relevant documents such as legislation, government policy, journal articles, grey literature, news articles and relevant websites that contained information on taxation policies applied to foods. The key documentation type was legislation, specifically Acts. The other forms of documentation were collected as a means of identifying the existence of food taxation policies, but where possible, the legislation behind these policies was located. A ledger was kept to record the search process.

The search terms used for each approach were tailored to the search engine or approach being used. For example, search terms for the PacLII database for each PICT were ‘customs duty’ OR ‘customs levy’ OR ‘customs tariff’ OR ‘excise’ OR ‘excise tax’ OR ‘import duty’ OR ‘import levy’ OR ‘import tariff’ OR ‘tariff’ (see online Supplementary Material for further information).

Key informants and stakeholders from PICTs were engaged to seek additional information, enquire about any missing data or inconsistencies and assess the completeness of data collection. We contacted nineteen key informants, who were a mix of government officials, health advisors and academics identified from government websites, the authors’ networks and referrals. Some provided legislation and other confirmed that the main data sources had been found.

Search results were screened against the inclusion criteria by EW. Some documents were lengthy and thus were screened using keyword searching, such as: ‘customs tariff’, ‘customs levy’, ‘customs duty’, ‘excise tax’, ‘excise duty’, ‘excise’, ‘import duty’, ‘import levy’ and ‘import tariff’.

### Policy identification

Following broad eligibility screening, individual taxation policies were scrutinised to determine if they met the inclusion criteria. For taxation policies that met all inclusion criteria, the relevant data were extracted into a data extraction table. When information on a taxation policy was incomplete, two or more sources of reference to the policy were necessary before the policy could be included, and these policies were a focus of engagement with key informants to determine their accuracy.

### Data extraction

Data about each policy, including key characteristics, were extracted in Microsoft Excel and analysed. Data that were extracted included: the jurisdiction of the taxation policy, the date of implementation or the removal of the tax, the type of tax (excise tax, import tariff, etc), the tax level (in local currency), the tax design (ad valorem, volumetric, etc), the specific food groups the tax had been applied to, any apparent motivation or justification for the tax and any evaluations of the policy that were found during the search process.

**Fig. 2:**
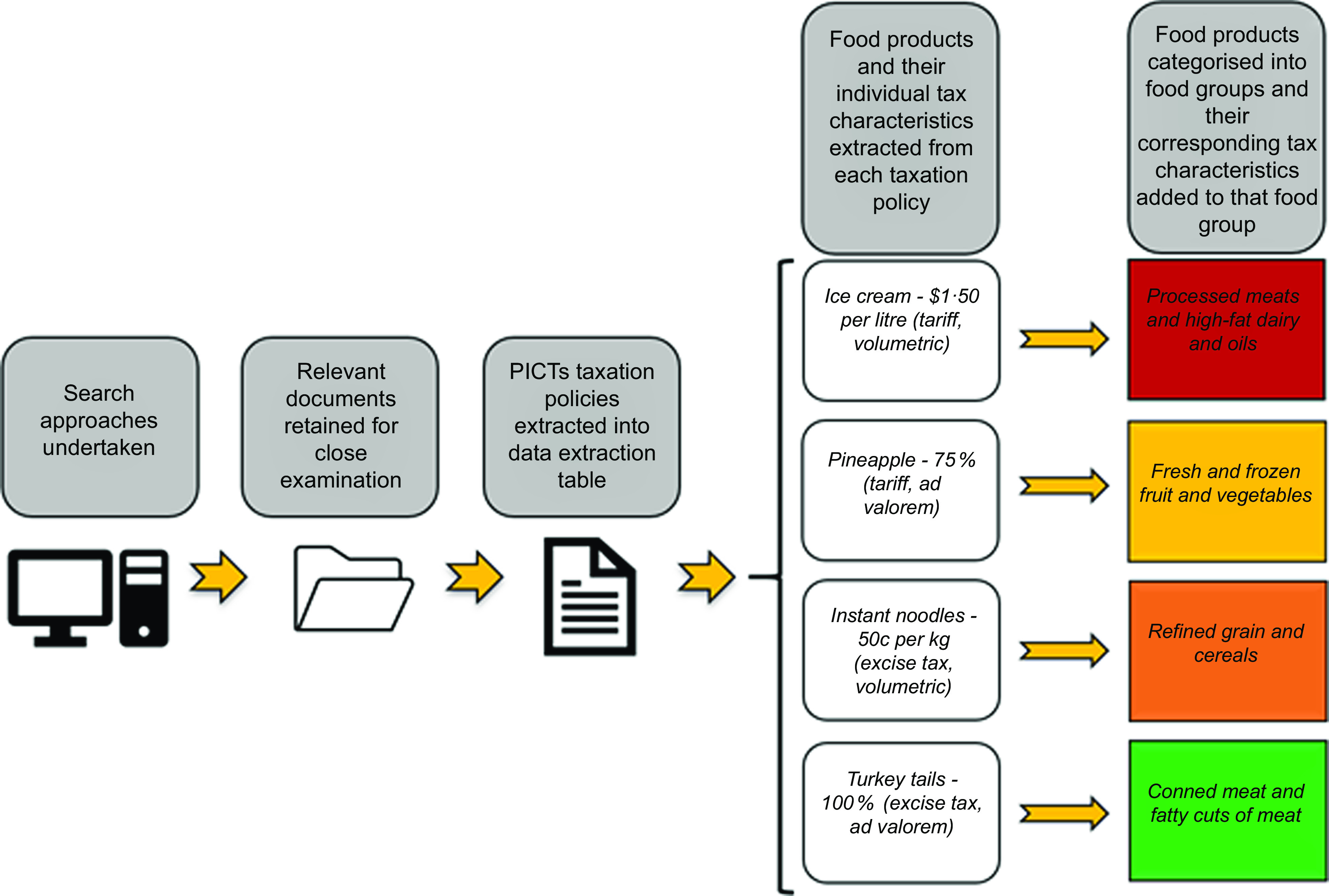
Extraction process for systematic review of food tax policies. PICTs, Pacific Island Countries and Territories

For further clarification of the data extraction process, and the difference between the taxation policy and the individual taxes applied to foods and subsequently food groups, see Fig. 2.

Tariffs were defined as measures applied to imported goods at their point of entry into a jurisdiction and not applied to domestically produced goods. Import duties, excise duties (applying to imports only) and import levies were included under the term tariff given the similarities between these mechanisms. Excise taxes were defined as being applied to domestically produced and imported products.

### Analysis

The analysis consisted of directed content analysis of the policies according to characteristics of interest. Taxation policies were categorised into excise taxes and tariff policies, then further separated into individual taxes by food group. Due to the volume of individual food products in the documentation, foods were collated into nine main food groups for reporting based on the nutrition guidelines developed by the Pacific Community (SPC), which specifically pertain to a Pacific diet. These food groups were whole grains and carbohydrate-dense vegetables; refined grain and cereals; processed foods, sugar, and salt; fresh and frozen fruit and vegetables; canned fruit and canned vegetables with low salt or sugar; dried fruit and high-sodium processed vegetables; lean proteins; canned meat and fatty cuts of meat and processed meats and high-fat dairy and oils. Beverages were excluded because they have been reviewed elsewhere^(18)^.

## Results

**Fig. 3:**
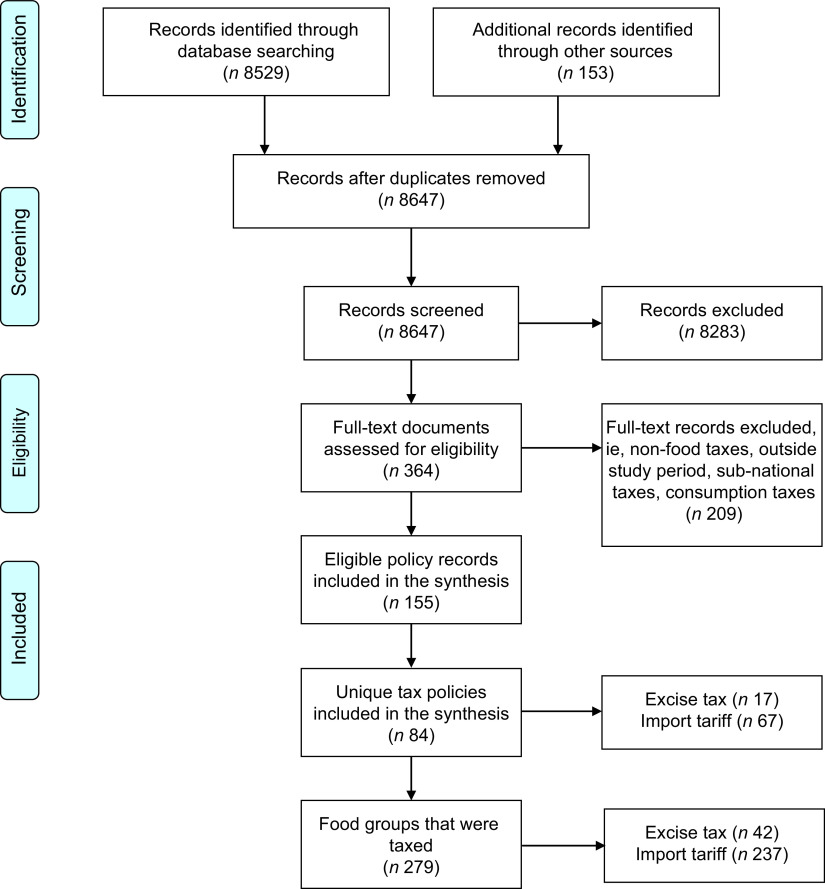
PRISMA flow diagram, with the number of taxation policies, excise taxes and tariff policies and individual taxes. From Moher D, Liberati A, Tetzlaff J, Altman DG, The PRISMA Group (2009). Preferred Reporting Items for Systematic Reviews and Meta-Analyses: The PRISMA Statement. PLoS Med 6(6): e1000097

From the search, 8682 documents were selected for screening, including legislation, reports, academic literature and news articles. Of these, 155 information sources met the study’s inclusion criteria and constitute the data examined in these results (Fig. 3).

**Fig. 4:**
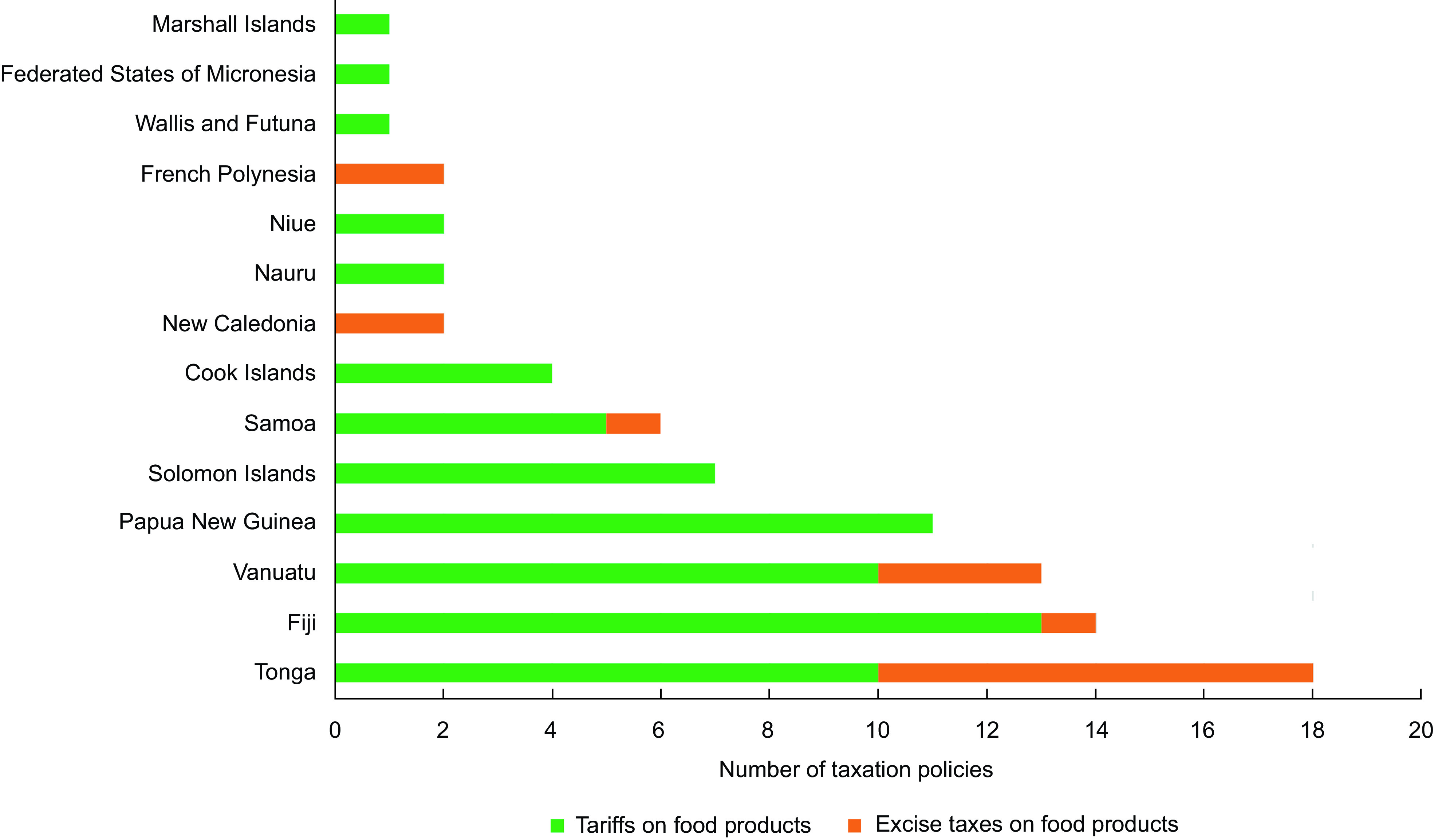
Number of food taxation policies in Pacific Island Countries and Territories by tax type (2000–2020)

During the study period, two-thirds of PICTs (fourteen of twenty two) implemented taxation policies on food that met the study definition (Table 2) and one-quarter of PICTs introduced excise taxes on food (six of twenty two). Figure 3 shows the number of tariffs and excise taxes from the taxation policies, and Fig. 4 shows the number of tariffs and excise taxes in each of the fourteen PICTs with taxation policies. Only four PICTs implemented excise taxes and tariffs, twelve PICTs implemented only tariffs and two implemented only excise taxes. For those PICTs with both types of taxation policies, sometimes one tax type would replace the other (e.g. the implementation of an excise tax saw the lowering of the tariff rate on that same food item) and some tax types were applied simultaneously on food items.

**Table 2:** Pacific Island Country and Territories with and without identified food tax policies

Excise tax policies	Tariff policies	No food tax policies
Fiji	Cook Islands	American Samoa
French Polynesia	Federated States of Micronesia	Northern Mariana Islands
New Caledonia	Fiji	Guam
Samoa	French Polynesia	Kiribati
Tonga	Marshall Islands	Palau
Vanuatu	Nauru	Pitcairn Islands
	New Caledonia	Tokelau
	Niue	Tuvalu
	Papua New Guinea	
	Samoa	
	Solomon Islands	
	Tonga	
	Vanuatu	
	Wallis and Futuna	

At the policy level, there were seventeen excise taxation policies and sixty-seven tariff policies introduced during the study period, giving a total of eighty-four unique food taxation policies. The total number of new policies per year peaked in 2018 with nine policies implemented.

### Food group

The eighty-four food taxation policies corresponded to a total of 279 tax changes targeting specific food groups. Although tariffs were applied to foods from all food groups, excise taxes were only applied to food from five of the nine food groups.

### Tariffs applied

**Fig. 5:**
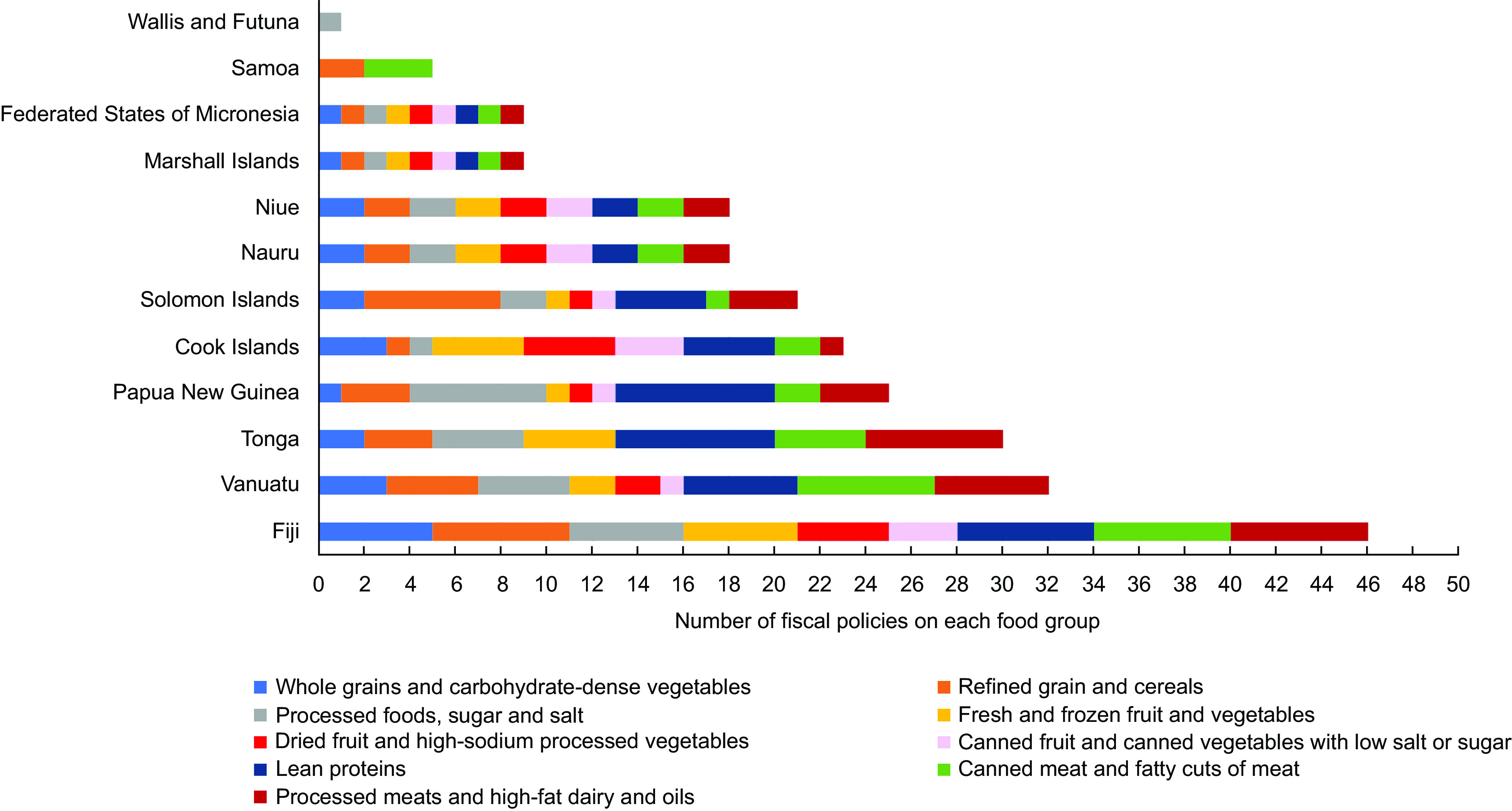
Number of individual tariffs implemented on each food group by Pacific Island Country and Territory (2000–2020)

Figure 5 depicts the number of tariffs applied to each food group (*n* 237) for each of the twelve PICTs with a tariff policy. Tariffs in a single policy generally covered most or all the food groups. Tariff taxation policies appeared to occur where a PICTs would provide a comprehensive update of tariff rates or changes across a broad range of goods at one time, or completely revise an entire tariff schedule. Tariff changes were distributed throughout the study period. Most PICTs had at least one tariff change applied to every food group during the study period. Just over half of import tariffs (138 of 237, 58 %) were applied to less healthy food groups (i.e. refined grain and cereals; processed foods, sugar and salt; dried fruit and high-sodium processed vegetables; canned meat and fatty cuts of meat and processed meats and high-fat dairy and oils) (Fig. 5). This pattern suggests that on average, tariff changes do not appear to target any particular food groups, except for the three PICTs that applied tariffs to specific foods.

### Tariff rates

Across the PICTs, there was a wide range in tariff rates, ranging from 0 % to 300 % ad valorem (Table 3). The most common type of tariff was ad valorem, whereas volumetric tariffs were only occasionally applied.

**Table 3:** Tariff rate range in each food group by Pacific Island Country or Territory (introduced or modified from 2000 to 2020)

Jurisdiction	Whole grains and carbohydrate-dense vegetables	Refined grain and cereals	Processed foods, sugar and salt	Fresh and frozen fruit and vegetables	Dried fruit and high-sodium processed vegetables	Canned fruit and canned vegetables with low salt or sugar	Lean proteins	Canned meat and fatty cuts of meat	Processed meats and high-fat dairy and oils
Cook Islands	0–75 %	–	–	0–75 %	0–75 %	0–75 %	0–75 %	–	–
Fiji	0–32 % and 4·45 FJD per kg	0–32 % and 1·48 FJD per kg	0–42 %	0–32 %	0–27 %	0–32 %	0–32 %	0–32 %	0–32 %
Marshall Islands	0 %	0–5 %	5 %	0 %	5 %	0–5 %	0 %	0–5 %	5 %
F. States of Micronesia	3 %	3 %	3 %	3 %	3 %	3 %	3–25 %	3 %	3 %
Nauru	7–10 %	7–30 %	30 %	7–10 %	30 %	7–30 %	7–10 %	7–10 %	7–30 %
Niue	0–25 %	0–25 %	0–25 %	0–25 %	0–25 %	0–25 %	0–25 %	0–25 %	0–25 %
Papua New Guinea	15–25 %	0–25 % and 110 PGK – 124 PGK per tonne	0–82 %	15–25 %	15–25 %	15–25 %	0–25 % and 1·60 PGK – 3·50 PGK per kg	15–25 %	0–25 %
Samoa	–	0 %	–	–	–	–	–	10–300 %	–
Solomon Islands	0–10 %	0–10 % and 0·20 SBD per kg	0–10 %	10 %	10 %	10 %	0–10 %	10 %	0–10 %
Tonga	0–15 %	0 %	0–20 %	0–20 %	–	–	0–20 %	0–15 %	0–15 %
Vanuatu	10–30 %	0–20 % and 20 VT per kg	10–30 % and 20 VT per kg	30 %	20–30 %	20 %	0–60 %	20–55 % and 20 VT per kg	0–40 % and 20–22 VT per L or kg
Wallis and Futuna	–	–	10 %	–	–	–	–	–	–

FJD, Fijian Dollar; PGK, Papua New Guinea Kina; SBD, Solomon Islands Dollar; VT, Vanuatu Vatu.

A dash denotes no tariff identified for that PICTs food group.

Trade appeared to have a meaningful impact on tariff changes in PICTs. Among the PICTs that had multiple tariff changes, tariff rates generally decreased during the study period. Trade agreements generally impose maximum tariff rates for certain products, and depending on their terms, may require tariffs to be reduced or other existing policies to be modified.

Two-thirds of tariff changes (159 of the 237) were in jurisdictions that are World Trade Organization members: Fiji (*n* 46 tariffs), Vanuatu (*n* 32), Tonga (*n* 30 policies), Papua New Guinea (*n* 25), Solomon Islands (*n* 21) and Samoa (*n* 3). World Trade Organization membership imposes some constraints on a country’s ability to increase tariffs above bound levels (as well as imposing limits on other policies, such as import bans or domestic taxes that discriminate between domestically produced and imported food). Samoa’s accession to the World Trade Organization required the removal of the turkey tail import ban. As a negotiated concession, a tariff rate was applied at 300 % for the first year, reduced to 100 % after two years, and then further reduced over time^(19)^. An analysis in Vanuatu has reported how World Trade Organization commitments have affected the availability, nutritional quality and accessibility of food^(20)^. Several regional trade agreements also exist within the Pacific^(15)^, including the Pacific Agreement for Closer Economic Relations (now Pacific Agreement for Closer Economic Relations plus after its revision)^(21)^ and the Pacific Island Countries Trade Agreement^(22)^. Pacific Island Countries Trade Agreement has been expressly identified as a reason for the lowering of tariff rates for the Cook Islands and Niue^(23–25)^.

Public health objectives also appeared to influence some tariff changes. Some countries appeared to have applied high rates uniformly across most food groups, whereas other countries appeared to have targeted specific products or food groups with a high rate. The food groups of canned meats and fatty cuts of meat, and lean proteins appeared to be most consistently the target of high rates, but most food groups had reasonable variation in the highest tariff rates applied to them. Of the twenty-two PICTs, three PICTs applied tariff changes to specific foods rather than across all foods at the same time (Wallis and Futuna, Samoa, and Tonga). These tariffs were applied to foods in the following food groups: processed foods, sugar and salt; refined grains and cereals; canned meat and fatty cuts of meat and were likely increased for health reasons. Another example is Fiji’s 2012 increase in palm oil tariffs (from 15 % to 32 %). This increase was expressly introduced to reduce consumption and promote better health outcomes. An evaluation of this policy showed that the tariff increase led to a subsequent reduction in palm oil imports^(26,27)^. Another case study in Fiji demonstrated that a reduction in tariffs on fruit and vegetables resulted in an increase in imports of fruit and vegetables^(28)^. There were also examples of tariff policies, however, that may discourage healthy nutrition, e.g. high tariff rates on fish and seafood to protect the local fishing industry.

### Excise taxes applied

**Fig. 6:**
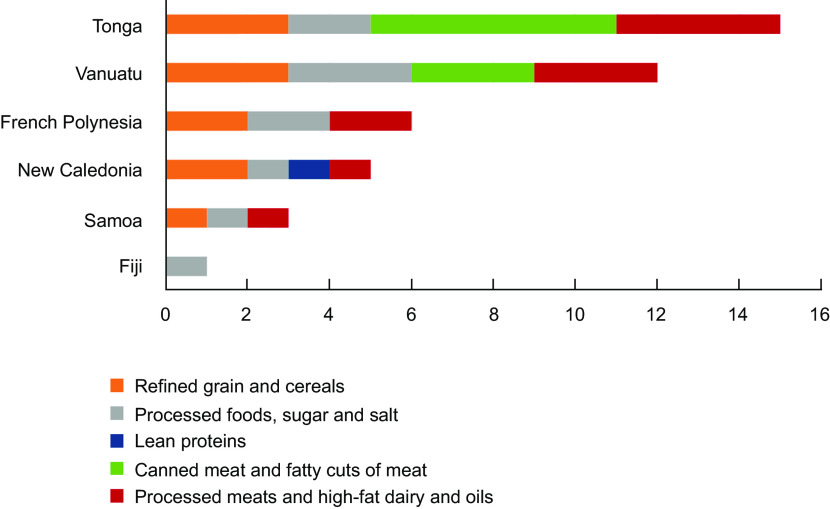
Number of excise taxes implemented on each food group by Pacific Island Country and Territories (2000–2020)

Figure 6 shows the number of excise taxes applied to each food group for each of the six PICTs that had an excise tax change. Excise taxes targeted less healthy food groups (i.e. forty one of forty two of food groups were less healthy foods, Fig. 6). Processed foods, sugar and salt were consistently subject to excise, while other popular food groups targeted included refined grains and cereals, processed meats and high-fat dairy and oils and canned meat and fatty cuts of meat. All three food groups with a focus on fruits and vegetables did not have any excise taxes, nor did the whole grains and carbohydrate-dense vegetables group. Examples of the individual foods that were taxed within the food groups included instant noodles, cakes, biscuits, mutton flaps, canned and preserved fish, sugar, pasta and white rice. Targeting of these unhealthy foods is very likely to have been implemented for health reasons.

The number of excise taxes on food increased during the study period. Excise taxes were introduced earliest in French Polynesia (2001, with changes in 2019), but they were introduced more recently in Vanuatu (2010, 2012 and 2014), Tonga (2013–2018), Samoa (2016) and New Caledonia (2018 and 2019).

### Excise tax rates

Unlike tariffs, most food excise taxes identified in this study resulted from entirely new taxes, rather than changes in tax rates. In one setting, excise taxes were reversed, i.e. when Fiji removed sugar from the Excise Tax Act Schedule^(29)^. As with tariffs, there was considerable variation in the excise tax rate, including within food groups. Table 4 presents the upper and lower excise tax rates from 2000 to 2020 for each PICT, by food group. The excise taxes introduced ranged from up to 8 % in Samoa and 22 % in New Caledonia and up to TOP 5/kg in Tonga (US$2·10), CFP 120/kg in French Polynesia (US$1·10) and VT 20/kg in Vanuatu (US$0·17). Most excise tax designs were volumetric, with some ad valorem and tiered tax designs. French Polynesia applied a broadly applicable tiered volumetric tax to foods based on their sugar content or volume of sugar. The foods included in this excise tax were products such as biscuits, ice cream, jams and chocolate.

**Table 4:** Excise tax rate range in each food group by Pacific Island Country or Territory (introduced or modified from 2000 to 2020)

Jurisdiction	Refined grain and cereals	Processed foods, sugar and salt	Canned meat and fatty cuts of meat	Processed meats and high-fat dairy and oils	Lean proteins
Fiji	–	Sugar removed from the Excise Act Schedule	–	–	–
French Polynesia	Gradient 1 0–4·99 g O FCFP per kg or L	Gradient 1: 0–4·99 g O FCFP per kg or L	–	Gradient 1 0–4·99 g O FCFP per kg or L	–
	Gradient 2 5–9·99 g 20–40 FCFP per kg or L	Gradient 2 5–9·99 g 20–40 FCFP per kg or L		Gradient 2 5–9·99 g 20–40 FCFP per kg or L	
	Gradient 3 10–29·99 g 40–60 FCFP per kg or L	Gradient 3 10–29·99 g 40–60 FCFP per kg or L		Gradient 3 10–29·99 g 40–60 FCFP per kg or L	
	Gradient 4 30–39·99 g 60–90 FCFP per kg or L	Gradient 4 30–39·99 g 60–90 FCFP per kg or L		Gradient 4 30–39·99 g 60–90 FCFP per kg or L	
	Gradient 5 40 g and + 85–120 FCFP per kg or L	Gradient 5 40 g and + 85–120 FCFP per kg or L and 20 FCFP per net kg*		Gradient 5 40 g and + 85–120 FCFP per kg or L	
New Caledonia	22 % and 140–150 FCFP per kg	22 %	–	22 %	22 %
Samoa	8 %	5 %			–
Tonga	0·25–2 TOP per kg	2–5 TOP per kg	0·40–2 TOP per kg	0·25–2·50 TOP per kg	–
Vanuatu	20 VT per kg	20 VT per kg	20 VT per kg	20 VT per kg	–

FCFP, Central Pacific franc; TOP, Tongan Pa’anga; VT, Vanuatu Vatu.

A dash denotes no tax identified for that PICTs food group.

* Pure sugar had a set rate per net kilogram, whereas the other rates were set on the specific weight of food products.

### Food groups with taxation policies

Of the nine food groups, five appeared to have been specifically targeted for tariff and excise policies. These groups were refined grain and cereals; processed meats and high-fat dairy and oils; lean proteins; canned meat and fatty cuts of meat and processed foods, sugar and salt. The taxed products in these groups were often listed among the foods that the SPC recommends individuals eat less or least of^(30)^. Of the excise taxes, none were applied to products in any of the fruit and vegetable food groups, and tariffs were also less common in this group. Altogether, these patterns suggest that among PICTs with food excise taxes, these are likely to have been applied for health reasons.

## Discussion

Food taxes are commonly used in the Pacific. This review identifies food taxation policy changes in fourteen of twenty-two PICTs between 2000 and 2020, with a total of eighty four such policies introduced or modified.

There has been an increasing movement in the region towards introducing excise taxes on unhealthy food, with five PICTs introducing this policy from 2000 to 2020, in line with recommendations by the WHO to improve food environments^(16)^. Excise taxes specifically targeted unhealthy food products (such as lamb flaps and instant noodles in Tonga). Some excise taxes have a sizable level and encompass a breadth of unhealthy foods, suggesting likely promising effects for NCD prevention and health outcomes.

In addition, import tariffs are commonly used in the Pacific for revenue collection, trade protection and sometimes for health reasons. Import tariff rates generally reduced over time and are often on declining schedules in response to trade commitments, with varying rates by country of origin. In many Pacific jurisdictions, import tariffs apply to a large proportion of the food that is consumed^(5)^.

### Strengths and limitations of this study

This study has identified food taxation policies introduced in PICTs from 2000 to 2020 through a comprehensive search process. The search process was robust and included multiple avenues for data gathering to provide assurance that the data in this study is accurate and comprehensive. The findings obtained for this study reinforce and build upon existing research, by providing a clear picture of food-specific taxation policies in the PICTs over the review period and providing new information that has not yet been reported in other food taxation policy studies in the Pacific^(7,26)^. A strength of this study is that it provides further detail about the characteristics of food taxation policies within the PICTs, such as which foods and food groups the taxes are applied to and in which jurisdictions. It also highlights key features of the excise taxes and tariffs, such as the rate and design of the taxes.

This study obtained almost all data via databases and relied on documentation that has been published online. Due to possible resource limitations among the small jurisdictions in the Pacific, regular updates may not have been made to these databases, resulting in some data gaps. However, due to the multiple data search avenues, data was often triangulated through more than one source, which has strengthened the validity of the findings.

Further research could explore the impacts of food taxes in the Pacific and the factors that facilitated their implementation (like those studies conducted previously evaluating food taxes)^(31–33)^. We were not able to explicitly examine rationale of tax changes because this information was not consistently available from legislation and other collected data, but in some cases this is available elsewhere^(34)^.

### Implications

Excise taxes applied to both imported products and any domestic production are recommended for NCD prevention. Consistency of excise tax rates between imported and domestically produced foods can reduce substitution to equally unhealthy domestically produced products^(35)^. Excise taxes are less vulnerable to changes required by trade agreements (e.g. removal of turkey tail ban in Samoa and subsequent reduction in tariffs)^(19)^. Accordingly, such policies may be more comprehensive and effective than tariffs in reducing the consumption of the foods they are applied to. This is an area where food taxation policy could be expanded in the Pacific and elsewhere to further pursue health objectives.

The effectiveness of excise taxes in improving health outcomes and reducing NCDs (while minimising negative externalities) is dependent on their design. For example, excise taxes will be most effective in PICTs where they are applied to foods that are deemed key contributors to NCDs in PICTs^(36–38)^. The use of a Pacific-oriented nutrient profiling tool to systematically identify food products appropriate for excise taxation could be an effective method to achieve this^(39)^. Excise taxes should preferably be nutrient specific (applied to sugar, salt or fat content) rather than ad valorem (applied to a product’s value) or volumetric (applied to a product’s volume or weight)^(40)^. This is because nutrient-specific tax designs are more effective in reducing the quantity of unhealthy components in food products (and in turn reducing consumption of these components), while also reducing the taxation burden on populations and (in turn) contributing to ensuring equity for lower-income groups^(35)^. Volumetric taxes on food are also preferable to ad valorem taxes, given their greater impact on bodyweight reduction, while reducing a possible regressive tax burden^(41)^. Nutrient and volumetric taxes require regular adjustment with inflation to maintain real value in line with inflation^(42)^ and effectiveness, and may be more challenging to implement.

When taxing any food, an assessment of the equity impacts of taxing food groups without viable alternatives is important (e.g. taxing certain meat products may have a negative impact on equity if these products are also a key protein sources for certain populations)^(33)^. Combined policies that increase tax on less healthy food and decrease tax on healthy food are likely to promote healthy substitution and promote equity. The investment of tax revenue into greater health spending can also be designed to improve equity.

### Conclusions

Food taxes are used throughout the Pacific to pursue what appear to be a range of policy objectives. From 2000 to 2020, one-quarter of PICTs introduced excise taxes that specifically targeted unhealthy foods and are likely to be effective in improving health outcomes. There are opportunities to introduce or strengthen food taxation policies to respond to the NCD crisis in the Pacific including greater adoption of tiered or nutrient-specific excise taxes that are systematically applied to unhealthy foods.

## Supporting information

Walby et al. supplementary material Walby et al. supplementary material

## Supplementary material

For supplementary material accompanying this paper visit https://doi.org/10.1017/S1368980023002914
