# Supplementary material for: Food tax policies in Pacific Island Countries and Territories: systematic policy review
Source: Public Health Nutr. 2023 Dec 21;27(1):e20. doi: 10.1017/S1368980023002914 (PMC10830374; doi:10.1017/S1368980023002914)
Supplement: Walby et al. supplementary material [file S1368980023002914sup001.docx]

# Supplementary material

For manuscript titled *Food tax policies in Pacific Island Countries and Territories: systematic policy review.*

**Further search strategy methods**

1. **Pacific Islands Legal Information Institute**

The Pacific Islands Legal Information Institute (PacLII) database was the first search engine searched for legislation. The search term string used for each PICT was: "customs duty" OR "customs levy" OR "customs tariff" OR "excise" OR "excise tax" OR "import duty" OR "import levy" OR "import tariff" OR "tariff".

All search results were examined for relevance against the inclusion criteria. A document search for keywords showing relevant data was undertaken for every document if it was unclear whether the policy met the inclusion criteria. The keywords used for this were: “customs tariff”, “customs levy”, “customs duty”, “excise tax”, “excise duty”, “excise”, “import duty”, “import levy” and “import tariff”.

1. **Large Search Engines**

Broad search engines of Google, Google Scholar, Factiva newspaper database, and Scopus were searched in the second approach. A consistent approach was used across the search engines.

The applied search terms used Boolean Logic and the string included: taxation OR tax OR taxes OR tariff* OR excise OR "excise tax" OR "import duty" OR "customs duty" OR "customs levy" OR "customs tariff" OR "import tariff*" OR "import lev*" OR “food tax*” OR “health tax*” OR "junk food tax" OR "fat tax" AND “[insert PICT name here]”. Due to the high search volume yields, only the first fifty results were examined for each search engine. For all search engines, all links were opened, and the contents of the documentation was examined for potential inclusion data. If the document was longer than one page, the document was searched to establish whether the source contained relevant inclusion data. Keywords were entered using the find function. These were: “tax”, “tariff”, “import duty”, “import levy”, “customs duty”, “customs levy”, “excise”, “junk food tax”, “fat tax”, “health tax”.

1. **PICTs Government Websites and Repositories**

Searching of all available PICTs government websites and their legislation repositories for taxation documentation was conducted as the third approach. Most of the repositories had basic search functions, and searching was tailored to each PICT’s website. A basic search approach was devised dependant on whether there was a search function available. The following search term string was used where possible: "customs duty" OR "customs levy" OR "customs tariff" OR "excise" OR "excise tax" OR "import duty" OR "import levy" OR "import tariff" OR "tariff". Where searching using a search bar was not possible, the titles of legislation were reviewed against the keywords: “tax”, “tariff”, “customs”, “import” and “excise”.

Documents of relevance were opened and the title and contents section headings were read to determine contents. A document search for keywords showing relevant data was undertaken for every document if the inclusion was still unclear. The keywords used for this were: “customs tariff”, “customs levy”, “customs duty”, “excise tax”, “excise duty”, “excise”, “import duty”, “import levy” and “import tariff”.

1. **Key Informants and Stakeholders**

This last search approach engaged key informants and stakeholders from PICTs to collate further information. This approach was used to provide additional information about data inconsistencies or missing data, or to provide confirmation that all main data sources had been already found. Contact with key informants was conducted in the first instance via email. When documents or taxation policy information were received via email with key informants, it was recorded in the data extraction table.

Information gathered from this search approach was included in the study up until the first full draft of the results was completed, at which point any further correspondence was not included.
